# Procedural sedation and analgesia versus general anesthesia for hysteroscopic myomectomy: A cost‐effectiveness analysis alongside a randomized controlled trial

**DOI:** 10.1111/aogs.70053

**Published:** 2025-10-18

**Authors:** Julia F. van der Meulen, Mohamed El Alili, Sjors F. P. J. Coppus, Helen S. Kok, Jaklien C. Leemans, Marlies Y. Bongers, Judith E. Bosmans

**Affiliations:** ^1^ Department of Obstetrics and Gynecology Máxima Medical Centre Veldhoven The Netherlands; ^2^ Grow School for Oncology and Reproduction Maastricht University Maastricht The Netherlands; ^3^ Department of Health Sciences, Faculty of Science Vrije Universiteit Amsterdam, Amsterdam Public Health Research Institute Amsterdam The Netherlands; ^4^ Department of Obstetrics & Gynecology Alrijne Ziekenhuis Leiden The Netherlands

**Keywords:** cost‐effectiveness analysis, fibroids, hysteroscopic myomectomy, outpatient setting, procedural sedation and analgesia with propofol

## Abstract

**Introduction:**

Hysteroscopic myomectomy is the first‐choice treatment for symptomatic type 0 and 1 fibroids and was traditionally performed under general anesthesia. Over the last decade, surgical procedures have increasingly been performed in an outpatient setting under procedural sedation and analgesia. However, studies evaluating the safety and cost‐effectiveness of hysteroscopic myomectomy under procedural sedation and analgesia are lacking. This study aimed to assess the cost‐effectiveness of procedural sedation and analgesia with propofol in an outpatient setting for hysteroscopic myomectomy compared to general anesthesia in an operating room.

**Material and Methods:**

This was a cost‐effectiveness analysis from a societal perspective alongside a multicenter randomized controlled non‐inferiority trial. It was conducted in 14 Dutch university and teaching hospitals. Women aged ≥18 years with symptomatic type 0/1 fibroids (maximum number 3, maximum diameter 3.5 cm), sufficient knowledge of Dutch/English, and American Society of Anesthesiologists class 1/2 were included. A total of 209 women were randomized to hysteroscopic myomectomy with procedural sedation and analgesia in an outpatient setting (*n* = 106) or general anesthesia in an operating room (*n* = 103). The primary outcome of the clinical trial was the percentage of complete resections measured by transvaginal ultrasonography 6 weeks postoperatively (non‐inferiority margin 7.5% of incomplete resections). Societal costs and quality‐adjusted life years (QALYs) were assessed. Societal costs were related to the percentage of complete resections and QALYs. Incremental Cost‐Effectiveness Ratios (ICERs) were calculated. Uncertainty surrounding these was estimated using bootstrapping. Follow‐up period was 12 months. Dutch Trial Register NTR 5357.

**Results:**

Hysteroscopic resection was complete in 86/98 women (87.8%) with procedural sedation and analgesia and 79/89 women (88.8%) with general anesthesia, mean difference −0.0052 (95% CI −0.097 to 0.086). Non‐inferiority could not be demonstrated. There was a statistically significant difference in costs between procedural sedation and analgesia and general anesthesia (€−2577, 95% CI −3950 to −1157), but not in QALYs (0.011, 95% CI −0.019 to 0.040). The ICER per additional complete resection was €498 797 and for QALYs the ICER showed that procedural sedation and analgesia was dominant over general anesthesia.

**Conclusions:**

In this study, procedural sedation and analgesia for hysteroscopic myomectomy in an outpatient setting is cost‐effective compared to general anesthesia in an operating room, although non‐inferiority for complete resections could not be demonstrated. We therefore suggest the outpatient use of procedural sedation and analgesia for hysteroscopic myomectomy.

## Graphical abstract

Data on cost‐effectiveness of procedural sedation and analgesia for hysteroscopic myomectomy was lacking. Procedural sedation and analgesia for hysteroscopic myomectomy in an outpatient setting is cost‐effective compared to general anesthesia in an operating room. It should be considered to perform hysteroscopic myomectomy in an outpatient setting with procedural sedation and anesthesia.

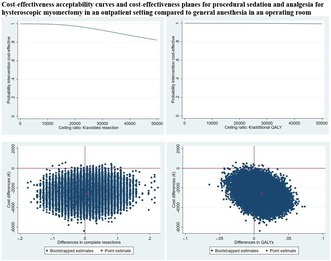

**Abbreviations**
ICER: Incremental cost‐effectiveness ratio
QALY: Quality‐adjusted life year
RCT: Randomized controlled trial

**Key message**

Procedural sedation and analgesia for hysteroscopic myomectomy in an outpatient setting is cost‐effective compared to general anesthesia in an operating room. It should be considered to perform hysteroscopic myomectomy in an outpatient setting with procedural sedation and anesthesia.

## 1 INTRODUCTION

Uterine fibroids are a frequent finding in premenopausal women.^1^ Fibroids can have a negative influence on quality of life by causing symptoms like heavy menstrual bleeding, dysmenorrhea, and abdominal pain.^2^ In addition, fibroids place a considerable economic burden on society: a systematic review estimated the annual total costs associated with fibroids in the United States to be $5.9 to $34.4 billion. Costs included healthcare and lost productivity costs and costs arising from fibroid‐related obstetric complications.^3^

Hysteroscopic myomectomy is the treatment of first choice for submucosal fibroids. Generally, this procedure was performed in an operating room with general or regional anesthesia.^4^ However, over the last decades, the development of smaller diameter hysteroscopic instruments has enabled a shift in gynecologic surgery from the operating room in a clinical setting with general anesthesia to an outpatient setting with local or no anesthesia.^5^, ^6^, ^7^ Cost‐effectiveness of diagnostic hysteroscopies and hysteroscopic polyp removal in an outpatient compared to inpatient setting has been demonstrated.^8^, ^9^ However, when larger diameter instruments are required (for procedures such as hysteroscopic myomectomy), the need for cervical dilatation could result in patient's discomfort, leading to reduced patient satisfaction, lower acceptability, and higher incompleteness rates when no or local anesthesia is used.^10^, ^11^

Procedural sedation and analgesia with propofol can be used to accomplish moderate to deep sedation for patients undergoing painful or unpleasant procedures.^12^, ^13^ Hence, procedural sedation and analgesia could enable patients to undergo gynecologic procedures in an outpatient setting if local anesthesia is insufficient. Procedural sedation and analgesia have been described for hysteroscopic procedures, endometrial ablation, vaginal prolapse surgery, and laparoscopic procedures.^14^ However, patient numbers in these studies are small, and large randomized controlled trials (RCT) are lacking.

Literature on the use of procedural sedation and analgesia for hysteroscopic myomectomy is missing. Therefore, we performed a cost‐effectiveness analysis alongside a multicenter non‐inferiority RCT comparing procedural sedation and analgesia with general anesthesia for hysteroscopic myomectomy (PROSECCO trial). We hypothesized that procedural sedation and analgesia could enable the performance of hysteroscopic myomectomy in an outpatient setting, leading to shorter admission time, faster recovery, and return to work, hereby reducing costs involved. Conversely, procedural sedation and analgesia compared to general anesthesia could result in higher patient discomfort, leading to higher rates of incomplete procedures and the need for surgical reinterventions.

Results of the non‐inferiority analysis and other clinical outcomes have been published elsewhere.^15^ This study assessed the cost‐effectiveness of procedural sedation and analgesia compared to general anesthesia for hysteroscopic myomectomy.

## 2 MATERIAL AND METHODS

### 2.1 Study design

This cost‐effectiveness analysis was performed alongside a multicenter non‐inferiority RCT (PROSECCO trial) in 14 Dutch university and teaching hospitals. The complete study protocol was published before.^16^ The study was registered prospectively in the Dutch Trial Register (NTR 5357‐registration date: August 11, 2015) and the ethics committee of the Máxima Medical Centre granted ethical approval.

Women aged ≥18 years with a maximum of 3 symptomatic type 0 or 1 fibroids with a maximum diameter of 3.5 cm were eligible for inclusion. Furthermore, they had to be American Society of Anesthesiologists (ASA) class 1 or 2 and have sufficient knowledge of the Dutch or English language. Exclusion criteria were the presence of known clotting disorders or severe anemia (Hb <5.0 mmol/L).

After written informed consent was obtained, women were randomized between hysteroscopic myomectomy with procedural sedation and analgesia or hysteroscopic myomectomy with general anesthesia. Follow‐up period was 12 months.

### 2.2 Interventions

Hysteroscopic myomectomy was conducted by a qualified gynecologist with a resectoscope or morcellation device. Procedural sedation and analgesia were Non‐Anesthesiologist Administered Propofol (NAAP) sedation, in which propofol was administered intravenously as a sedative and remifentanil or alfentanil as an analgesic. According to the Dutch guidelines and safety requirements, procedural sedation and analgesia was administered and monitored by a qualified sedation practitioner (an anesthetic nurse with additional qualifications to administer procedural sedation and analgesia).^17^, ^18^ General anesthesia was inhalational or total intravenously, with the use of a laryngeal mask or endotracheal tube.

### 2.3 Outcomes

The primary outcome of the clinical trial was the percentage of complete resections as measured by transvaginal ultrasonography 6 weeks postoperatively by a sonographer blinded to the surgical outcome or received intervention. Hysteroscopic myomectomy was considered complete when no signs of an intracavitary remnant of the resected fibroid(s) were present.

Secondary outcomes were societal costs and quality of life (EQ‐5D‐5L questionnaire).^19^ Details and results of all other outcomes of this trial were published elsewhere.^15^, ^16^

The EQ‐5D‐5L questionnaire assesses quality of life on five dimensions (mobility, selfcare, activities of daily living, pain/discomfort, and depression/anxiety). Health states were converted to utility scores using the Dutch tariff.^19^ Using the area‐under‐the‐curve method, Quality‐Adjusted Life Years (QALYs) were calculated by multiplying the amount of time a participant spent in a specific health state by the associated utility score. Transitions between health states were linearly interpolated.

Costs were measured from a societal perspective (healthcare costs, home care costs, and lost productivity costs) using the Institute for Medical Technology Assessment's (iMTA) Medical Consumption Questionnaire (iMCQ) and the iMTA Productivity Cost Questionnaire (iPCQ).^20^, ^21^ These self‐reported questionnaires were filled out by participants at 8 weeks, 6 months, and 12 months of follow‐up.

Healthcare utilization was valued using standard costs from the Dutch costing guideline.^22^ Medication costs were valued using prices from www.medicijnkosten.nl, last accessed on September 23, 2021. Lost productivity costs due to absenteeism from work were calculated according to the friction cost approach using gender‐specific income values of the Dutch population. This means that it is assumed that an employee is replaced after 108 days of sickness leave, which is based on the number of filled vacancies and open vacancies from Statistics Netherlands in line with recommendations from the Dutch costing guideline.^22^, ^23^

Intervention costs were based on fixed cost prices for each of the three intervention options (general anesthesia in operating room, procedural sedation and analgesia in outpatient setting or procedural sedation and analgesia in operating room). These cost prices were estimated using a bottom‐up micro‐costing approach, including costs related to the time a patient spent in the operating theater and recovery room, which was measured during the PROSECCO trial.^16^ It was assumed that procedural sedation and analgesia were administered in an outpatient setting and model of care and general anesthesia in an operating room in an ambulatory model of care, based on the definitions as described in the international consensus statement for recommended terminology describing hysteroscopic procedures.^24^ In addition, the time medical specialists (gynecologist and anesthetist) and supporting staff (operating assistant, recovery nurse, nurse, physician assistant) spent on the procedure was taken into account. Dutch official salary tables were used to estimate the costs per minute per type of human resource associated with the procedure, in line with recommendations from the Dutch costing guideline.^22^ Finally, costs related to anesthesia were included, which were based on expert opinion. For procedural sedation and analgesia, anesthesia consisted of an anesthetic and analgesic (i.e., propofol and alfentanil) and a nasal oxygen cannula. For general anesthesia, this consisted of an anesthetic and analgesic (i.e., propofol and sufentanil), a mask with filter, and a laryngeal mask. The bottom‐up calculation of the treatment costs for the different types of treatments is included in Table S1. All costs were expressed in Euros using consumer price indices for the year prior to the year of analysis (2020).^23^ Discounting was not necessary because follow‐up was restricted to 12 months.

### 2.4 Statistical analysis

Based on literature and expert opinion, the expected incidence of incomplete resections was estimated as 2.5%.^25^, ^26^, ^27^ The non‐inferiority margin was set at a risk difference of 7.5% incomplete resections. With an alpha chosen as 0.025 and allowing for a drop‐out of 10% due to loss to follow‐up, 206 women needed to participate to achieve 90% power.

The cost‐effectiveness analyses were conducted according to the intention‐to‐treat principle. Missing data were imputed using Multiple Imputation with Chained Equations (MICE).^28^ Cost and effect data were assumed to be missing at random, meaning that missing observations are explained by observed variables.^29^ The imputation model included outcome variables and predictor variables that either differed at baseline, were related to missing data, or were associated with the outcome (see Table 1 for included variables). To account for the skewed distribution of cost data, predictive mean matching was used in MICE.^30^ The number of imputed data sets was increased until the loss of efficiency was <5%, resulting in 10 imputed data sets.^30^ Each of the imputed data sets was analyzed separately as described below, and results were pooled using Rubin's rules.^31^

**TABLE 1:** Multiply imputed effects and costs for hysteroscopic myomectomy with procedural sedation and analgesia or general anesthesia.

Outcomes	Intervention group (procedural sedation and analgesia) *N* = 106	Control group (general anesthesia) *N* = 103	Mean difference (95% CI)^a^
	Mean (SE)		
Complete resection	0.88 (0.033)	0.88 (0.033)	−0.0052 (−0.097 to 0.087)
QALY	0.94 (0.011)	0.93 (0.010)	0.011 (−0.020 to 0.041)
Healthcare costs			
Primary care	257 (35)	490 (143)	−233 (−677 to −34)
Secondary care	240 (29)	316 (48)	−76 (−195 to 16)
Medication	1.29 (0.34)	1.84 (0.76)	−0.55 (−2.11 to 0.48)
Home care	0 (0)	62 (47)	−62 (−223 to −16)
Hospital	345 (89)	847 (225)	−502 (−1092 to −151)
Other centra	1.66 (1.66)	1.88 (1.88)	−0.22 (−5 to 5)
Treatment^b^	452 (NA)	1111 (NA)	−659 (NA)
Total healthcare costs	1297 (113)	2829 (328)	−1532 (−2322 to −994)
Patient costs			
Travel costs	17 (2.23)	25 (4.57)	−9 (−19 to −1)
Lost productivity costs			
Absenteeism FCM	1386 (362)	2033 (595)	−647 (−1513 to 273)
Absenteeism HCA	1603 (460)	2164 (665)	−561 (−1632 to 635)
Presenteeism	102 (74)	551 (190)	−448 (−771 to −173)
Unpaid help	379 (178)	320 (97)	59 (−184 to 690)
Total lost productivity costs FCM	1867 (441)	2903 (594)	−1036 (−2070 to 120)
Total lost productivity costs HCA	2084 (535)	3035 (663)	−951 (−2163 to 463)
Total healthcare costs	1297 (113)	2829 (328)	−1532 (−2322 to −994)
Total societal costs (FCM)	3181 (506)	5757 (661)	−2577 (−3973 to −1126)
Total societal costs (HCA)	3398 (607)	5889 (719)	−2491 (4015 to −805)

*Note*: Multiple imputation model consisted of variables that differed at baseline, were related to missing data or were associated with the outcome: weight, use of hormonal medication, myoma type, size of myoma, other myomas and the number of other myomas. The imputation procedure was stratified for treatment arm.

Abbreviations: 95% CI, 95% confidence interval; FCM, Friction cost method; HCA, Human capital approach; QALY, Quality adjusted life‐year; SE, standard error.

^a^ Uncertainty around cost differences estimated using the non‐parametric bootstrap with 5000 replications (bias‐corrected and accelerated intervals).

^b^ Intervention costs were based on a fixed cost price meaning that costs per patient were equal for all patients within a group. Therefore, 95% confidence intervals cannot be estimated for intervention costs.

Societal costs were related to the percentage of complete resections and QALYs. Seemingly unrelated regression models were used to estimate incremental costs and effects between treatment groups, which account for the correlation between costs and effects.^32^

Incremental Cost‐Effectiveness Ratios (ICERs) were calculated by dividing the incremental costs by incremental effects. Bias‐corrected and accelerated bootstrapping was used to estimate statistical uncertainty (5000 replications). Statistical uncertainty surrounding ICERs was illustrated by plotting the bootstrapped cost‐effect pairs on a cost‐effectiveness plane (CE plane). Cost‐Effectiveness Acceptability Curves (CEACs) were also estimated, demonstrating the probability that the intervention is cost‐effective compared to usual care for a range of different ceiling ratios (i.e., the willingness‐to‐pay threshold for one point effect extra).^33^ CEACs were estimated using the parametric *p*‐value approach for Incremental Net‐Monetary Benefits (INMBs).^34^ In the Netherlands, the generally used willingness‐to‐pay threshold for healthcare interventions ranges between €20 000 and €80 000 per QALY gained.^35^ For outcome measures such as an additional complete resection, no willingness‐to‐pay threshold has been determined. Analyses were performed in StataSE 16® (StataCorp LP, CollegeStation, TX, US).

### 2.5 Sensitivity analyses

To assess the robustness of the results, four sensitivity analyses were performed. First, the economic evaluation was performed from the healthcare perspective (SA1), which included only healthcare costs. Second, the human capital approach was used to calculate lost productivity costs, including all lost productivity hours in the cost estimates (SA2). Third, a scenario where the intervention (procedural sedation and analgesia) was delivered in an operating room was assumed (SA3). In this scenario, treatment costs for the procedural sedation and analgesia group were higher than in the main analysis due to the use of the operating theater. Finally, a best–worst case scenario was evaluated for the clinical outcome complete resections (SA4). In the best‐case analysis, we assumed that the resection was complete for participants for whom data on this outcome was missing. Alternatively, in the worst‐case analysis, we assumed that the resection was incomplete in this group.

## 3 RESULTS

### 3.1 Participants

In total, 209 women were allocated to hysteroscopic myomectomy with procedural sedation and analgesia (*n* = 106) or general anesthesia (*n* = 103). Baseline characteristics of women and fibroids are presented in Table 2.

**TABLE 2:** Patient and fibroid baseline characteristics.

Characteristic	Procedural sedation and analgesia *N* = 106	General anesthesia *N* = 103
Age, years (mean, SD)	45.1 (6.4)	45.0 (7.7)
BMI (median, IQR)	24.7 (22.0–28.6)	25.7 (23.4–30.5)
Parity (median, IQR)	1 (0–2)	1 (0–2)
Previous uterine surgery	18 (17.0%)	24 (23.3%)
Specification		
Hysteroscopic myomectomy	8/18 (44.4%)	14/24 (58.3%)
Other	10/18 (55.6%)	10/24 (41.7%)
Use of hormonal medication at the time of surgery	52 (49.1%)	45 (43.7%)
Reason for hysteroscopic myomectomy^a^		
Abnormal uterine bleeding	101 (96.2%)	96 (93.2%)
Abdominal complaints	6 (5.7%)	9 (8.7%)
Subfertility	4 (3.8%)	5 (4.9%)
Other	1 (1.0%)	2 (1.9%)
No. submucous myomas intended to be resected	119	113
One	93 (87.7%)	94 (91.3%)
Two	13 (12.3%)	8 (7.8%)
Three	0 (0.0%)	1 (1.0%)
Myoma type		
Type 0 (normal)	46 (39.3%)	56 (52.8%)
Type 1	71 (60.7%)	50 (47.2%)
Maximum size (diameter [mm]‐mean, SD)	20.9 (6.4)	19.9 (6.8)
Presence of additional fibroids that can't be resected hysteroscopically		
Yes	36 (34.6%)	30 (29.1%)

*Note*: Percentages are column percentages based on the number of observations available.

Abbreviations: BMI, Body mass index; IQR, Interquartile range; No., number; SD, Standard deviation.

^a^ Multiple reasons can apply.

In four women, intracavitary fibroids were absent during surgery. Hence, hysteroscopic myomectomy was not performed. Three additional women were recruited to compensate for the unexpected drop‐out (total *n* = 209). In the procedural sedation and analgesia group, 101/106 patients (95.3%) received the allocated intervention versus 95/103 patients (92.2%) in the general anesthesia group. Reasons for this are presented in the study flow diagram, Figure S1. Conversion from procedural sedation and analgesia to general anesthesia during the procedure did not occur. The primary outcome was missing in 8/106 women (7.5%) in the procedural sedation and analgesia group and 14/103 women (13.6%) in the general anesthesia group.

### 3.2 Primary clinical outcome

Complete hysteroscopic resection was achieved in 86/98 (87.8%) women with procedural sedation and analgesia and in 79/89 (88.8%) with general anesthesia, mean difference −0.0052 (95% CI −0.097 to 0.087). Significant non‐inferiority could not be demonstrated as described elsewhere.

### 3.3 Costs

An overview of the costs in both treatment groups is presented in Table 1. Costs in the procedural sedation and analgesia group were statistically significantly lower than in the general anesthesia group (mean difference €−2577, 95% CI −3973 to −1126). The main contributors to these lower costs were intervention‐related costs (€−659) and hospital visits (€−502, 95% CI −1092 to −151). Although absenteeism costs according to both the friction cost method (€−647, 95% CI −1513 to 273) and the human capital approach (€−561, 95% CI −1632 to 635) were notably lower in the procedural sedation and analgesia group compared to the general anesthesia group, these differences were not statistically significant. However, presenteeism costs (€−448, 95% CI −771 to −173) were statistically significantly lower with procedural sedation and analgesia vs. general anesthesia.

### 3.4 Cost‐effectiveness analysis

Results of the cost‐effectiveness analysis are presented in Table 3. The ICER for complete resections was €498 797, indicating that on average the additional costs per additional complete resection are €498 797 in the general anesthesia group compared to the procedural sedation and analgesia group. This large ICER is due to dividing the incremental costs by the very small difference in complete resections between the two groups.

**TABLE 3:** Results of the cost‐effectiveness analyses and sensitivity analyses.

Outcome	Costs (€)			Effects			ICER (€/unit of effect)	CE plane^b^			
	Intervention mean (SE)	Control mean (SE)	Difference (95% CI)^a^	Intervention mean (SE)	Control mean (SE)	Difference (95% CI)^a^		NE (%)	SE (%)	SW (%)	NW (%)
Main analysis: Societal perspective											
Complete resections	3181 (506)	5757 (661)	−2577 (−3950 to −1157)	0.88 (0.033)	0.88 (0.033)	−0.0052 (−0.097 to 0.086)	498 797	0	53	47	0
QALY	3181 (506)	5757 (661)	−2577 (−3950 to −1157)	0.94 (0.011)	0.93 (0.010)	0.011 (−0.019 to 0.040)	Dominant	0	77	23	0
SA1: Healthcare perspective											
Complete resections	1297 (113)	2829 (328)	−1532 (−2357 to −996)	0.88 (0.033)	0.88 (0.033)	−0.0052 (−0.097 to 0.086)	296 489	0	53	47	0
QALY	1297 (113)	2829 (328)	−1532 (−2357 to −996)	0.94 (0.011)	0.93 (0.010)	0.011 (−0.019 to 0.040)	Dominant	0	77	23	0
SA2: Human capital approach											
Complete resections	3398 (607)	5889 (719)	−2491 (−3999 to −815)	0.88 (0.033)	0.88 (0.033)	−0.0052 (−0.097 to 0.086)	482 195	0	53	47	0
QALY	3398 (607)	5889 (719)	−2491 (−3999 to −815)	0.94 (0.011)	0.93 (0.010)	0.011 (−0.019 to 0.040)	Dominant	0	77	23	0
SA3: Treatment costs POK in operating theater											
Complete resections	3516 (506)	5757 (661)	−2241 (−3614 to −821)	0.88 (0.033)	0.88 (0.033)	−0.0052 (−0.097 to 0.086)	433 740	0	53	47	0
QALY	3516 (506)	5757 (661)	−2241 (−3614 to −821)	0.94 (0.011)	0.93 (0.010)	0.011 (−0.019 to 0.040)	Dominant	0	77	23	0
SA4: Best–worst case scenario											
Best‐case scenario complete resections	3181 (506)	5757 (661)	−2577 (−3950 to −1157)	0.89 (0.030)	0.90 (0.029)	−0.016 (−0.099 to 0.067)	159 842	0	63	37	0
Worst‐case scenario complete resections	3181 (506)	5757 (661)	−2577 (−3950 to −1157)	0.81 (0.038)	0.77 (0.042)	0.044 (−0.067 to 0.16)	Dominant	0	22	78	0

Abbreviations: 95% CI, 95% confidence interval; CE plane, cost‐effectiveness plane; ICER, incremental cost‐effectiveness ratio; NE, North‐east quadrant; NW, North‐west quadrant; QALY, Quality adjusted life‐year; SA, sensitivity analysis; SE, South‐east quadrant; SW, South‐west quadrant.

^a^ Uncertainty around cost differences estimated using the non‐parametric bootstrap with 5000 replications (bias‐corrected and accelerated intervals).

^b^ Effects were inverted before being plotted on the CE‐plane.

**FIGURE 1:**
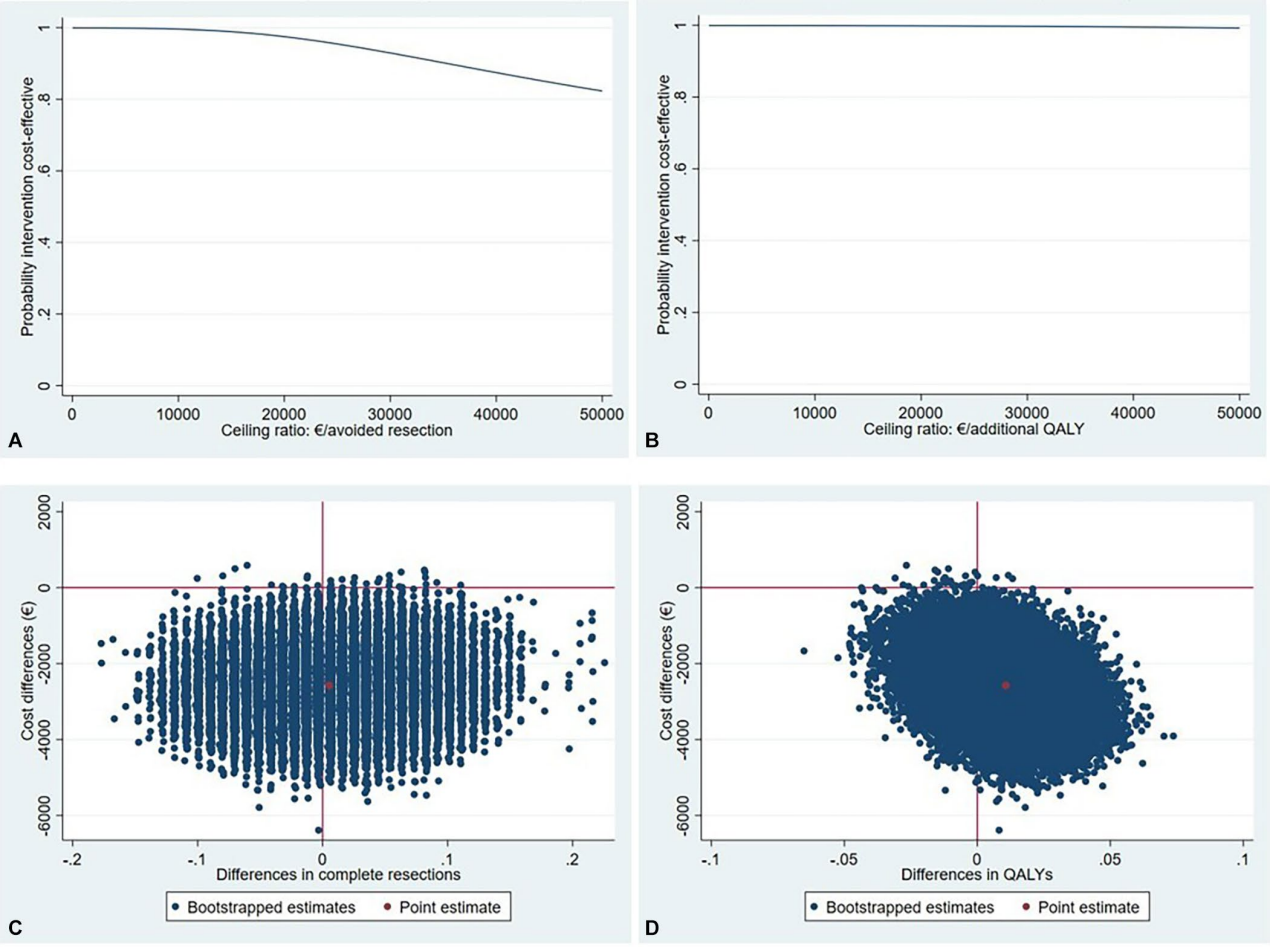
Cost‐effectiveness acceptability curves and cost‐effectiveness planes. (A) Cost‐effectiveness acceptability curve for complete resections. (B) Cost‐effectiveness acceptability curve for quality‐adjusted life years. (C) Cost‐effectiveness plane for complete resections. (D) Cost‐effectiveness plane for quality‐adjusted life years. QALY, Quality‐adjusted life years.

The CE plane shows that the bootstrapped cost‐effect pairs are equally divided across the southeast and the southwest quadrants of the plane, confirming no significant difference in complete resections and lower costs with procedural sedation and analgesia compared to general anesthesia (Figure 1C). The CEA curve (Figure 1A) shows that the probability that procedural sedation and analgesia is cost‐effective in comparison with general anesthesia is 1.00 at willingness‐to‐pay values between €0 and 10 000 per additional complete resection.

The difference in QALYs between the procedural sedation and analgesia and general anesthesia group was 0.011, which was not statistically significant (95% CI −0.019 to 0.040). The ICER for QALYs showed that for QALYs, procedural sedation and analgesia was dominant over general anesthesia (i.e., less expensive and more effective). This was also confirmed by the CE plane (Figure 1D) where the majority of bootstrapped cost‐effect pairs are located in the southeast quadrant. The CEA curve (Figure 1B) shows that the probability that procedural sedation and analgesia is cost‐effective in comparison with general anesthesia is 1.00, 1.00, and 0.99 at willingness‐to‐pay values of 0, 20 000, and 50 000 €/per QALY gained, respectively.

### 3.5 Sensitivity analyses

The results of the sensitivity analyses are in line with the results of the main analyses (Table 3). The largest impact on cost‐effectiveness results was found when the economic evaluation was performed from the healthcare perspective (SA1) in which the cost saving decreased from €2577 to €1532. Using the human capital approach to calculate lost productivity costs (SA2), or assuming that the intervention was delivered in an operating theater (SA3), had a minimal impact on the results. The best‐case scenario for complete resections resulted in a larger difference between procedural sedation and anesthesia and general anesthesia in favor of general anesthesia compared to the main analysis due to the higher rate of missing data on complete resections in the general anesthesia group. In the worst‐case scenario, the difference in the proportion of complete resections turned around and was in favor of the procedural sedation and anesthesia group. However, non‐inferiority could not be shown.

## 4 DISCUSSION

In this cost‐effectiveness analysis from a societal perspective, it was demonstrated that hysteroscopic myomectomy performed with procedural sedation and analgesia in an outpatient setting was less expensive (−€2577 (−3950 to −1157)) than with general anesthesia in an operating room over a 12‐month follow‐up period. Results for completeness of resection showed a marginal, non‐significant difference between both groups. For an additional complete resection in the general anesthesia group, the costs are €498 797 as compared to the procedural sedation and analgesia group. The ICER for QALYs indicated that procedural sedation and analgesia was dominant over general anesthesia. The results of the sensitivity analyses were in line with the results of the main analyses.

The PROSECCO trial is the first RCT comparing the clinical effectiveness, safety, and cost‐effectiveness of procedural sedation and analgesia with general anesthesia for hysteroscopic myomectomy. By combining patient‐level cost data collected over a 12‐month follow‐up period with a detailed bottom‐up calculation of costs, a reliable and extensive overview of costs is presented. The fact that this study was a multicenter trial adds to the generalizability of the results to other hospitals in the Dutch setting. In the Netherlands, not all hospitals have similar outpatient facilities. In some hospitals, procedural sedation and analgesia is administered in the operating room with an ambulatory model of care and not in a typical outpatient setting/model of care, for reasons such as available facilities or the required presence of an anesthesiologist nearby. On the one hand, this could make the procedure more expensive, due to the use of operating theater facilities. On the other hand, the benefits of procedural sedation and analgesia, such as quicker recovery, which reduce both healthcare and societal costs, are still present. To include this variety in our analysis, we performed a sensitivity analysis in which procedural sedation and analgesia was administered in the operating room in a more ambulatory model of care. Although slightly more expensive than procedural sedation and analgesia in an outpatient setting, the administration of procedural sedation and analgesia in the operating room still resulted in cost savings compared to general anesthesia in the operating room.

Our study has several limitations. Although our study assessed many clinical outcomes, we did not evaluate patient satisfaction in this trial. However, we did include clinical outcomes such as (fibroid‐related) quality of life, which were not significantly different between the two treatment groups.^15^ Second, we did not include costs for anesthesiologic preoperative assessment in our bottom‐up calculation of treatment costs. In the Netherlands, large practice varieties exist (varying from no separate pre‐assessment appointment to a physical screening at the anesthesiology outpatient clinic). Hence, it is difficult to determine one way of pre‐assessing patients in the procedural sedation and analgesia and in the general anesthesia group, which is why we decided to exclude these costs from the analysis. It could be hypothesized that in case of procedural sedation and analgesia, a shorter and less extensive pre‐assessment consultation would be sufficient compared to general anesthesia. This could have led to an even larger difference in costs between both treatment arms. Therefore, including these costs in our analyses would presumably not have changed the study's conclusions. Third, we did not include the costs for either the morcellation device or resectoscope (including reusable or disposable). This could have influenced the total overall costs involved. However, since randomization was stratified for resection technique, no significant differences in distribution of both resection techniques among both groups were to be expected, so it was expected not to influence the observed effect or study conclusion.

Finally, the 12‐month follow‐up of the trial may be considered insufficient. However, we do not expect that there are longer term impacts on costs and effects related to procedural sedation and analgesia and general anesthesia that we did not capture in the current study. The direct impacts of either setting (operation room or outpatient) and type of anesthesia (procedural or general sedation) are expected to occur within the first 3 months of follow‐up, whereas the potential residual symptoms of an incomplete resection and the associated increased healthcare utilization rates are included in the second 9 months of follow‐up. Therefore, we do not consider it necessary to use health‐economic modeling techniques to extrapolate our findings to a longer time horizon.

Willingness to pay thresholds for completeness of hysteroscopic myomectomy have not been established. However, the probability of cost‐effectiveness at a willingness to pay threshold of 0 € per unit of effect extra was 1.0 for both complete resection and QALYs.

This study showed a small difference in the number of complete resections in favor of the general anesthesia group. However, costs were statistically significantly lower in the procedural sedation and analgesia group compared to the general anesthesia group. Because of the small difference in effects, the resulting ICER was very large (the extra costs are €498 797 in the general anesthesia group as compared to procedural sedation and analgesia group per additional complete resection). Thus, this ICER has no real meaning. Based on the statistically significantly lower costs and a small, statistically non‐significant difference in complete resections, we conclude that procedural sedation and analgesia is cost‐effective compared to general anesthesia for hysteroscopic myomectomy.

The cost‐effectiveness of procedural sedation and analgesia versus general anesthesia for hysteroscopic myomectomy was not assessed before. However, Diwakar and Cooper et al. presented the results of the OPT trial, in which clinical success and cost‐effectiveness were studied for hysteroscopic polypectomy in an outpatient setting with no or local anesthesia versus an inpatient setting with general or regional anesthesia. They found that hysteroscopic polypectomy in an outpatient setting was non‐inferior and cost‐effective compared to the same procedure in an inpatient setting.^9^, ^11^

For polypectomy, smaller diameter hysteroscopic instruments can be used, resulting in less need for cervical dilatation and hence the possibility to perform this procedure with no or local anesthesia. This would often not be tolerable for patients undergoing a hysteroscopic myomectomy, which is why procedural sedation and analgesia were used in the outpatient setting. Although office hysteroscopic myomectomy with local or no anesthesia has been described for fibroids with a diameter of 1.5–2.0 cm, studies on this new development are still limited (especially at the start of the PROSECCO trial); patient numbers are small and this technique is not widely used yet.^4^, ^36^, ^37^ Therefore, we did not include a local anesthesia arm in our study. Clinical outcomes are therefore difficult to compare among the two studies. However, the finding that performing the treatment in an outpatient setting is less expensive compared to an inpatient setting with similar results for clinical outcomes in both settings is comparable to our study.

This study was performed alongside the PROSECCO trial, a multicenter non‐inferiority RCT. Although the percentage of complete resections was comparable among the procedural sedation and analgesia and general anesthesia groups, non‐inferiority could not be demonstrated (risk difference −1.01%, 95% CI −10.36 to 8.34), because the study was underpowered. However, no significant differences in other secondary outcomes such as safety or quality of life were found either.^15^ Moreover, this cost‐effectiveness analysis (being a traditional superiority analysis to evaluate costs and effects) showed that using procedural sedation and analgesia in an outpatient setting is cost‐effective compared to general anesthesia in the operating room. We therefore suggest the outpatient use of procedural sedation and analgesia for hysteroscopic myomectomy.

Although our study consisted of a wide range of clinical outcome measures, it is important to take patient satisfaction into account. It is known that incomplete fibroid resection does not always necessitate additional surgery.^38^ Thus, an incomplete procedure does not always mean that treatment is perceived as unsuccessful. Also, it would be interesting to evaluate patients experiences and preferences with procedural sedation and analgesia versus general anesthesia. For future research, it is therefore recommended to include patient satisfaction as a secondary outcome to define perceived treatment success. Secondly, this study focused only on the use of procedural sedation and analgesia with propofol as a sedative option. Future studies could investigate if other sedation options (such as lighter sedation or different analgesics) could contribute to a further reduction of costs with similar surgical effectiveness.

## 5 CONCLUSION

Although non‐inferiority for completeness of resection could not be demonstrated, this study shows that the use of procedural sedation and analgesia for hysteroscopic myomectomy in a Dutch outpatient setting leads to a considerable reduction of costs compared to general anesthesia in the operating room, with a marginal, non‐significant difference in completeness of resection rates. There were no significant differences in QALYs. Therefore, we conclude that procedural sedation and analgesia for hysteroscopic myomectomy in an outpatient setting is cost‐effective compared to general anesthesia in the operating room. The outcomes of this study help gynecologists and anesthesiologists to counsel women with type 0 and 1 fibroids requiring a hysteroscopic myomectomy. We therefore suggest the outpatient use of procedural sedation and analgesia for hysteroscopic myomectomy.

## AUTHOR CONTRIBUTIONS

All authors were involved with the conception and planning of the study. Carrying out was performed by Julia F. van der Meulen, Sjors F. P. J. Coppus, Helen S. Kok, Jaklien C. Leemans, and Marlies Y. Bongers. Julia F. van der Meulen and Mohamed El Alili performed the analysis (supervised by Judith Bosmans) and drafted the first version of the manuscript, which was critically reviewed and approved by all authors.

## FUNDING INFORMATION

This study was funded by The Dutch organization for Health Research and Development (ZonMW), a governmental funding organization. Grant number 843002603. Before receiving the grant, this study protocol was peer‐reviewed by ZonMW.

## CONFLICT OF INTEREST STATEMENT

The authors declare they have no conflict of interest.

## ETHICS STATEMENT

The study was conducted according to the principles of the Declaration of Helsinki, and ethical approval was granted by the ethics committee of the Máxima Medical Centre in Veldhoven, the Netherlands (registration number NL54779.015.15‐reference number 15.106) on December 14, 2015. Written informed consent was obtained from all patients before taking part. The study was registered prospectively in the Dutch Trial Register (NTR 5357) on August 11, 2015. The date of initial participant enrolment was February 18, 2016. https://trialsearch.who.int/Trial2.aspx?TrialID=NTR5357.

## Supporting information


Figure S1

Table S1
