# Supplementary figures and images for: Procedural sedation and analgesia versus general anesthesia for hysteroscopic myomectomy: A cost‐effectiveness analysis alongside a randomized controlled trial
Source: Acta Obstet Gynecol Scand. 2025 Oct 18;104(12):2320–30. doi: 10.1111/aogs.70053 (PMC12668803; doi:10.1111/aogs.70053)

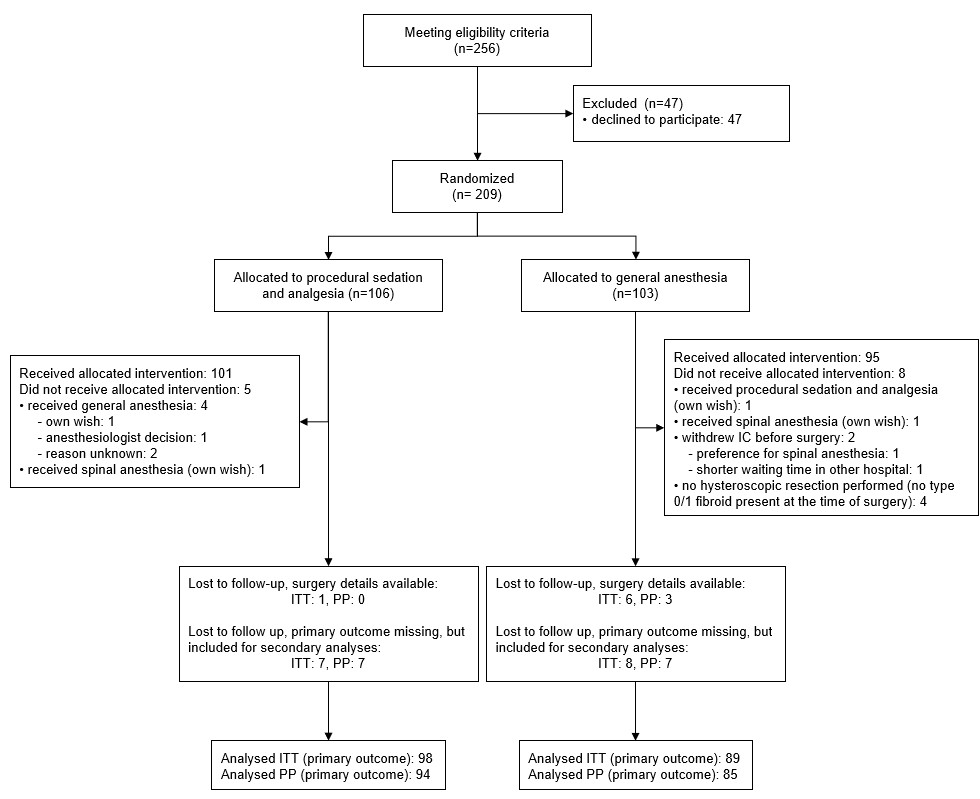

Supplement: Supplementary file 1 — Figure S1 [file AOGS-104-2320-s001.jpg]
