# Supplementary material for: Procedural sedation and analgesia versus general anesthesia for hysteroscopic myomectomy: A cost‐effectiveness analysis alongside a randomized controlled trial
Source: Acta Obstet Gynecol Scand. 2025 Oct 18;104(12):2320–30. doi: 10.1111/aogs.70053 (PMC12668803; doi:10.1111/aogs.70053)
Supplement: Supplementary file 2 — Table S1 [file AOGS-104-2320-s002.docx]

**Table S1. Bottom-up calculation of treatment costs**

|  | | **General anesthesia in operating room** | **Procedural sedation and analgesia in outpatient setting** | **Procedural sedation and analgesia in operating room** | **Source** | **Details** |
| --- | --- | --- | --- | --- | --- | --- |
| Procedure time (min) | | 48 | 41 | 41 | Trial data |  |
| Hospitalisation (min) | | 386 |  | 241 | Trial data |  |
| Recovery period (min) | | 45 | 60 | x | Expert opinion, trial data | General anesthesia in operating room: recovery in recovery room, supervised by recovery nurse.  Procedural sedation and analgesia in outpatient setting: recovery in outpatient setting, supervised by day-care nurse  Procedural sedation and analgesia in operating room: recovery in ward |
|  | |  |  |  |  |  |
| Costs for use of operating room / outpatient clinic | | 480,00 | 205,00 | 410,00 | Estimation based on rental prices for private operating rooms.^1^ | Operating room €10/min  Outpatient clinic €5/min  Procedure time x costs of operating room |
|  | |  |  |  |  |  |
| Personnel, costs / min | |  |  |  | Dutch costing guideline and official Dutch salary tables |  |
| Gynaecologist | 2,73 | 131,04 | 111,93 | 111,93 |  | Salary costs x procedure time  scale 5 |
| Anaesthetist | 2,73 | 89,76 | x | x |  | Salary costs x (procedure time-15)  scale 5 |
| Sedation practitioner | 0,94 | x | 38,54 | 38,54 |  | Salary costs x procedure time  FWG 60, scale 5 |
| Anaesthetic nurse | 0,83 | 39,84 | x | x |  | Salary costs x procedure time  FWG 55, scale 5 |
| Theatre nurse (scrub) | 0,83 | 39,84 | x | 34,03 |  | Salary costs x procedure time  FWG 55, scale 5 |
| Theatre nurse (circulation) | 0,83 | 39,84 | x | 34,03 |  | Salary costs x procedure time  FWG 55, scale 5 |
| Recovery nurse | 0,75 | 33,75 | x | x |  | Salary costs x recovery period  FWG 50, scale 5 |
| Day-care nurse | 0,65 | x | 39,00 | x |  | Salary costs x recovery period  FWG 45, scale 5 |
| Doctor’s assistant | 0,58 | x | 47,56 | x |  | Salary costs x procedure time x 2 (2 assistants per procedure)  Scale 4, level 5 |
| Total personnel costs | | 374,07 | 237,03 | 218,53 |  |  |
|  | |  |  |  |  |  |
| Anaesthetic costs | | 16,71 | 10,26 | 10,26 | Estimation based on expert opinion |  |
| Admission costs | | 240,06 |  | 149,57 | Trial data  Dutch costing guideline | Estimated costs for day care admission (8 hours): 298,52  Admission costs/ min: 0,62  Hospitalisation x admission costs |
| **Total costs** | | 1110,84 | 452,29 | 788,36 |  |  |

**Table S1. Bottom up calculation of treatment costs.** All prices are expressed in Euros. FWG: Functie Waardering Gezondheidszorg. ^1^Karim RB, Operatiekamer te huur. Medisch Contact. 2011;66(36): 2161-2163.
